# MicroRNA-224 is implicated in lung cancer pathogenesis through targeting caspase-3 and caspase-7

**DOI:** 10.18632/oncotarget.5224

**Published:** 2015-08-19

**Authors:** Ri Cui, Taewan Kim, Matteo Fassan, Wei Meng, Hui-Lung Sun, Young-Jun Jeon, Caterina Vicentini, Esmerina Tili, Yong Peng, Aldo Scarpa, Guang Liang, Yong Kui Zhang, Arnab Chakravarti, Carlo M. Croce

**Affiliations:** ^1^ Department of Molecular Virology, Immunology and Medical Genetics and Comprehensive Cancer Center, The Ohio State University, Columbus, OH, USA; ^2^ Department of Molecular and Cellular Oncology, The University of Texas MD Anderson Cancer Center, Houston, TX, USA; ^3^ Department of Medicine (DIMED), University of Padua, Padua, Italy; ^4^ Chemical Biology Research Center, School of Pharmaceutical Sciences, and Lung Cancer Research Center, The Zhoushan Hospital of Wenzhou Medical University, Wenzhou, Zhejiang, China; ^5^ Department of Radiation Oncology and Comprehensive Cancer Center, The Ohio State University, Columbus, OH, USA; ^6^ Applied Research on Cancer Network (ARC-NET) Research Centre, University of Verona, Verona, Italy; ^7^ Department of Anesthesiology, Wexner Medical Center, The Ohio State University, Columbus, OH, USA; ^8^ Division of Thoracic Surgery, State Key Laboratory of Biotherapy, West China Hospital, Sichuan University, Collaborative Innovation Center of Biotherapy, Chengdu, China; ^9^ Department of Pathology and Diagnostics, University of Verona, Verona, Italy; ^10^ Chemical Biology Research Center, School of Pharmaceutical Sciences, Wenzhou Medical University, Wenzhou, Zhejiang, China; ^11^ Department of Cardio-Thoracic Surgery, Lung Cancer Research Center, Zhoushan Hospital of Wenzhou Medical University, Zhoushan, Zhejiang, China

**Keywords:** miR-224, lung cancer, caspase-3, caspase-7

## Abstract

We recently reported that miR-224 was significantly up-regulated in non-small cell lung cancer (NSCLC) tissues, in particular in resected NSCLC metastasis. We further demonstrated that miR-224 functions as an oncogene in NSCLC by directly targeting TNFAIP1 and SMAD4. However, the biological functions of miR-224 in NSCLC are controversial and underlying mechanisms of miR-224 in the progression and metastasis of lung cancer remain to be further explored. Here we report that caspase3 (CASP3) and caspase7 (CASP7) are previously unidentified targets of miR-224 in NSCLC, and that miR-224 promotes lung cancer cells proliferation and migration in part by directly targeting CASP7 and down-regulating its expression. In addition, miR-224 attenuated TNF-α induced apoptosis by direct targeting of CASP3 resulting in reduction of cleaved PARP1 expression in lung cancer cells. Furthermore, the expression of miR-224 negatively correlates with the expression of CASP7 and CASP3 in tissue samples from patients with lung cancer. Finally, we found that activated NF-κB signaling is involved in the regulation of miR-224 expression in lung cancer. Our study provides new insight in understanding of oncogenic role of miR-224 in the lung cancer pathogenesis and suggests that NF-κB/miR-224/CASP3, 7 pathway could be a putative therapeutic target in lung cancer.

## INTRODUCTION

MicroRNAs (miRNAs) are small non-coding RNAs that repress the expression of target genes by inhibiting translation and/or stability of mRNAs [1]. Accumulated evidences indicate that miRNAs play a critical role in various biological processes including proliferation, differentiation, apoptosis, survival, development and metabolism [2-4]. Numerous miRNAs function as either tumor suppressors or oncogenes in a tissue specific manner, and the aberrant miRNAs expression is involved in the initiation and progression of human cancers [5]. To date, a number of lung cancer associated miRNAs have been reported. For instance, high expression of miR-155 and low expression of let-7a are associated with poor prognosis of lung cancer [6]. In addition, it has been reported that up-regulated miR-17-92 expression in lung cancer tissues [7] and increased miR-221 & 222 expression in aggressive NSCLC [8]. Recently, we also identified that miR-31 and miR-224 were significantly up-regulated in primary and metastasized NSCLC [9, 10].

MiR-224 has been reported to be up-regulated in several solid tumors including hepatocellular carcinoma [11, 12], colorectal cancer [13], breast cancer [14], and lung cancer [10], and repressing various targets such as API5, SMAD4, PHLPP1, PHLPP2, RKIP and TNFAIP1. On the other hand, miR-224 plays a tumor suppressive role in the prostate cancer by targeting TPD52 and/or TRIB1 [15, 16].

Both CASP3 and CASP7 are effector caspases which are activated by initiator caspases (caspase8 and 9) perform downstream execution steps of apoptosis by cleaving important cellular substrates [17]. CASP7 is highly related to CASP3, and these two caspases are activated during both death receptor- and mitochondria-induced apoptosis [18, 19]. Acquired chemo-resistance to apoptosis inducing anti-cancer drugs is frequently seen in CASP3 down-regulated cancer cells [20]. The down-regulation of CASP3 expression has been reported to be involved in lymph node metastases, poor overall prognosis and chemo-resistance of NSCLC [21, 22]. On the other hand, loss of CASP7 has been reported in colorectal cancer and gastric cancer [23, 24]. Mutations in *CASP7* are frequently found in cancer resulting in loss of its apoptotic function and contribute to the pathogenesis of certain types of human cancers [25]. Furthermore, aberrant modulation of CASP7 cleavage is thought to be critical factor involved in response of chemotherapies against breast and lung cancer cells [26]. A recent study demonstrated that CASP7 plays a key role in execution step of apoptosis in CASP3 deficient cancer cells suggesting that targeting of CASP7 could be an alternative therapeutic strategy for cancers with down-regulation of CASP3 [27].

Here, we show that miR-224 is involved in the lung cancer pathogenesis through direct targeting of CASP3 and CASP7. We also found that activated NF-κB signaling is involved in transcriptional regulation of miR-224 in NSCLC. Taken together, our study suggests that dysregulated NF-κB/miR-224/CASP3, 7 axis might have a critical role in the pathogenesis of lung cancer.

## RESULTS

### MiR-224 enhances proliferative and migratory ability of lung cancer cells

We recently reported that overexpression of miR-224 in NSCLC cell line, H1299, markedly enhanced proliferative and migratory effect of H1299 cells [10]. To overexpress miR-224, that would help us better determine its oncogenic function in other lung cancer cell lines, we transduced a Lenti-miR vector containing miR-224 precursor into NSCLC cell line, H3122 cells which express low level of miR-224 (Figure S1a). The vector containing miRZip-224 anti-miR-224 miRNA construct was transduced into H2228 cells, a NSCLC cell line which express high level of miR-224 (Figure S1a), to knockdown miR-224. The expressions of miR-224 after overexpression and/or knockdown were confirmed by qRT-PCR (Figure S1b and S1c). We measured the effects of miR-224 overexpression and inhibition, on proliferation and migration capabilities of respective cells. We found that in H3122 cells, the overexpression of miR-224 significantly enhanced cell proliferation and migration (Figure S2a and S2b). Conversely, in response to knockdown of miR-224, the proliferation and migration of H2228 cells were significantly reduced (Figure S2c and S2d). These results suggest, as expected, that in lung cancer cells, miR-224 exerts pro-migratory and proliferative functions.

### MiR-224 directly targets the 3′-UTR of CASP3 and CASP7

**Figure 1:**
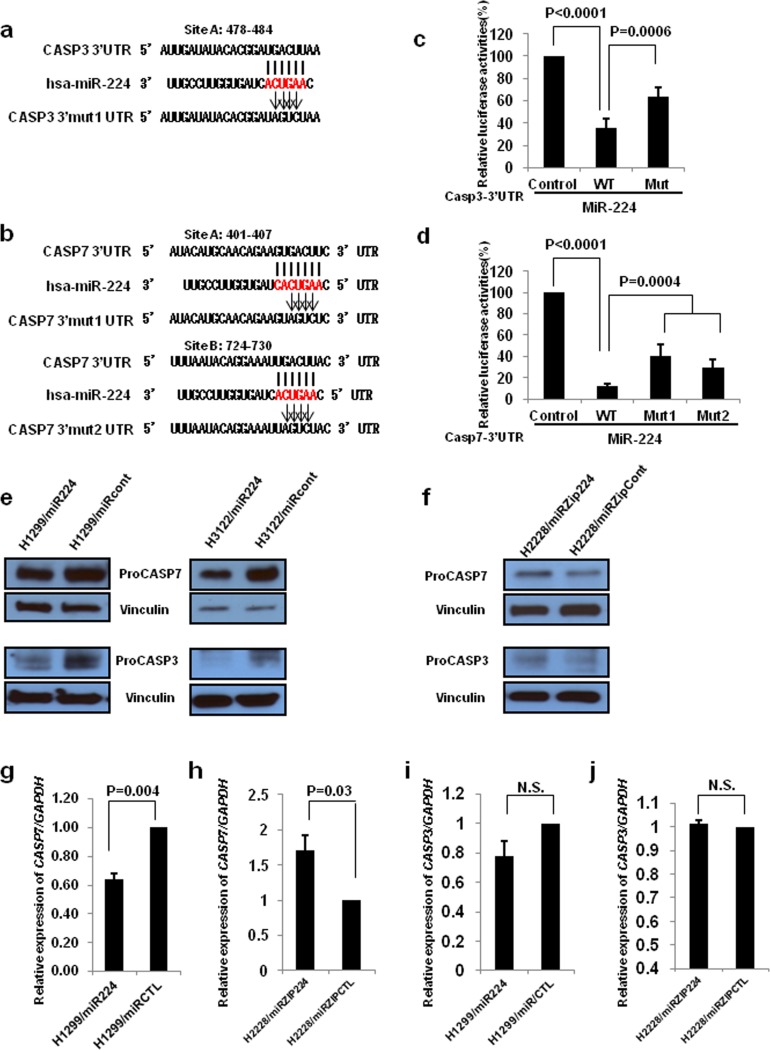
CASP3 and CASP7 are direct targets of *miR-224* **a.** and **b.** CASP3 **a.** and CASP7 **b.** 3′UTRs contain one and two predicted miR-224 binding sites. The alignments of seed region of miR-224 with CASP3 and CASP7 3′UTRs are shown. The arrows indicate the mutagenesis nucleotides. **c.** and **d.** Luciferase reporter constructs containing wild type or mutated CASP3 and CASP7 3′UTRs were co-transfected with miR-224 mimics into 293T cells. Luciferase reporter assays were contacted four times and data are presented as mean ± S.D. **e.** Western blot analysis to evaluate CASP3 and CASP7 protein levels in miR-224 overexpressing H1299 and H3122 lung cancer cells. **f.** Western blot analysis to evaluate CASP3 and CASP7 protein levels in miR-224 knockdown H2228 lung cancer cells. **g.** and **h.** qRT-PCR was conducted to measure CASP7 mRNA expression in miR-224 overexpressing **g.** or knockdown **h.** lung cancer cells. **i.** and **j.** qRT-PCR to measure CASP3 mRNA expression in miR-224 overexpressing **i.** or knockdown **j.** lung cancer cells. The values present mean ± S.D. as determined triplicated assays.

Our previous study demonstrated that miR-224 plays an oncogenic role by direct targeting of SMAD4 and TNFAIP1. To better understand the underlying mechanisms of miR-224 in lung cancer pathogenesis, we intend to find other plausible targets for miR-224 that could further explain its effects described in Figure S2. Bioinformatics analyses suggested that CASP3 and CASP7 are potential targets of miR-224, as the 3′UTRs of both transcripts contain sequences complementary to the miR-224 seed sequence (Figure 1a and 1b), which could explain the observed pro-survival effects of miR-224 in NSCLC. We found that co-transfection of each of 3′UTR and miR-224 mimics into 293T cells significantly reduced luciferase activity compared to the co-transfection of control vectors and miR-224 mimics. To validate target specificity, we mutated the binding site of miR-224 in their 3′UTRs, using QuickChange Mutagenesis kit. Of note, there is one predicted miR-224 binding site in the 3′UTR of *CASP3*, and two sites in the 3′UTR of *CASP7*. Co-transfection of miR-224 with the 3′UTR mutants of either CASP3 or CASP7 (*CASP3* 3′Mut UTR, *CASP7* 3′Mut1 UTR and *CASP7* 3′Mut2 UTR) significantly impaired the reduction capability of miR-224 on the luciferase activity of corresponding wild-type 3′UTRs (Figure 1c and 1d), suggesting that miR-224 negatively regulates CASP3 and CASP7 expressions by directly interacting with their 3′UTRs. Next, we investigated the effects of miR-224 on CASP3 and CASP7 at the mRNA and protein level using the cell lines we previously established and described on Figure S1. Overexpression of miR-224 remarkably reduced protein expression levels of both proCASP3 and proCASP7 in H1299 and H3122 cells, respectively (Figure 1e). Conversely, knockdown of miR-224 increased protein expression levels of proCASP3 and proCASP7 in H2228 cells (Figure 1f). In accordance with overexpression or knockdown of miR-224, CASP7 mRNA expression was significantly reduced or increased in lung cancer cells (Figure 1g and 1h). However, we observed no changes on CASP3 mRNA expression level in miR-224 overexpressing or knockdown lung cancer cells (Figure 1i and 1j) indicating miR-224 regulates CASP3 at translational levels. In summary, these results confirm that miR-224 directly targets CASP3 and CASP7 in lung cancer cells.

### CASP3 and CASP7 play crucial role in miR-224 mediated lung cancer progression

**Figure 2:**
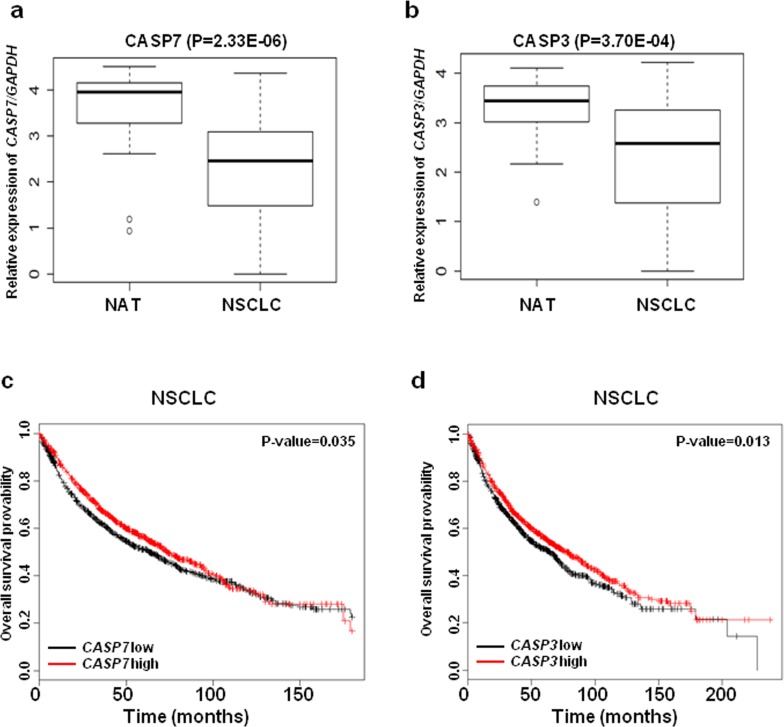
The expressions of CASP3 and CASP7 mRNA are down regulated in NSCLC **a.** CASP7 mRNA expressions in thirty paired NSCLC tissues and NATs. **b.** CASP3 mRNA expressions in thirty paired NSCLC tissues and NATs. GAPAH was used for normalization. **c.** and **d.** Kaplan-Meier plots of overall survival of lung cancer patients, stratified by expression of CASP7 (1926 patients) **c.** or CASP3 (1926 patients) **d.**. Data obtained from the Kaplan-Meier plotter database (kmplot.com/analysis).

**Figure 3:**
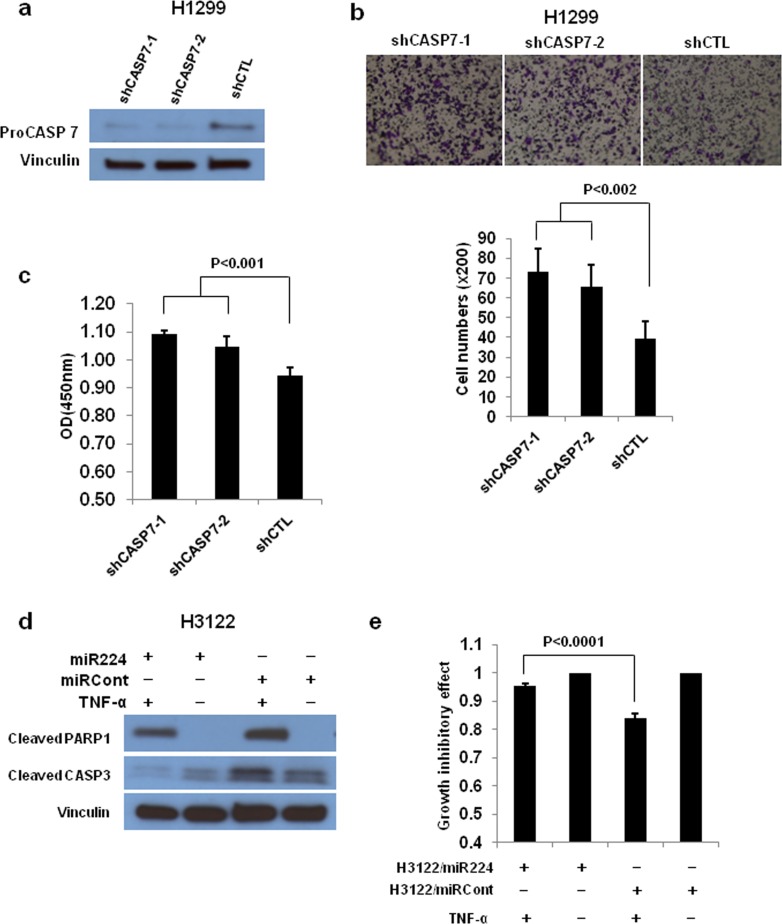
CASP3 and CASP7 have a essential role in miR-224 induced cell growth and migration **a.** Western blot analysis to determine CASP7 expression after knockdown of CASP7 by shCASP7. **b.** Cell migration assay for CASP7 knockdown H1299 cells using transwell membranes. Representative pictures were taken under 100 times magnification. The average counts were derived from six random microscopic fields. **c.** Cell proliferation assay for CASP7 knockdown H1299 cells. The cell growth rates were measured by cell counting kit 8. **d.** Western blot analysis to detect cleaved CASP3 and PARP1 in H3122/miR224 and H3122/miRCont cells treated with or without TNF-α for 4h. **e.** Cell proliferation assay for H3122/miR224 and H3122/miRCont cells treated with or without TNF-α for 24h. The values of cell proliferation assay present mean ± S.D. as determined by quintuplex assays.

CASP3 has an established role in lung cancer pathogenesis; however, the role of CASP7 in lung cancer remains largely unexplored. First, we examined the expression levels of CASP3 and CASP7 mRNA in thirty paired NSCLC tissues and normal adjacent tissues (NATs) from The Ohio State University (OSU) cohort. The expressions of CASP3 (3.70E-04) and CASP7 (2.33E-06) mRNA were significantly downregulated in NSCLC tissues compared to NATs (Figure 2a and 2b). We further analyzed the role of *CASP7* in the prognosis of lung cancer using a large public clinical microarray database [28] and found the existence of a trend toward improved overall survival in lung cancer patients with high expression of *CASP7* (Figure 2c). Same prognostic analysis were performed for *CASP3*, and we found that similarly to *CASP7* there is a correlation between high *CASP3* expression levels and overall survival of lung cancer patients (Figure 2d). To determine if there is a functional interaction between miR-244 and CASP7 activity, reflected in cell migration/proliferation outcome, we knocked down CASP7 in H1299 and H460 cells. Knockdown of CASP7 significantly increased migratory and proliferative ability of H1299 cells (Figure 3a-3c) and H460 cells (Figure S3a-S3c) indicating that miR-224 promotes lung cancer cell growth and migration at least partially by inhibiting CASP7. CASP3 is the most active effector caspase, which plays key role in execution step of apoptosis. To investigate whether miR-224 is involved in TNF-α induced cleavage of proCASP3, we treated H3122 cells with TNF-α. TNF-α treatment markedly induced cleaved CASP3 expression in H3122/miRCont cells but not in H3122/miR224 cells indicating that miR-224 might functionally prevent TNF-α induced apoptosis by inhibiting the expression of cleaved CASP3. We also found reduced levels of cleaved PARP1 in H3122/miR224 cells compared to H3122/miRCont cells after TNF-α treatment (Figure 3d). Further analyses showed that miR-224 significantly attenuated TNF-α induced cell growth inhibition in H3122 cells (Figure 3e). These results suggest that miR-224 is implicated in TNF-α induced apoptosis by direct targeting of CASP3.

**Figure 4:**
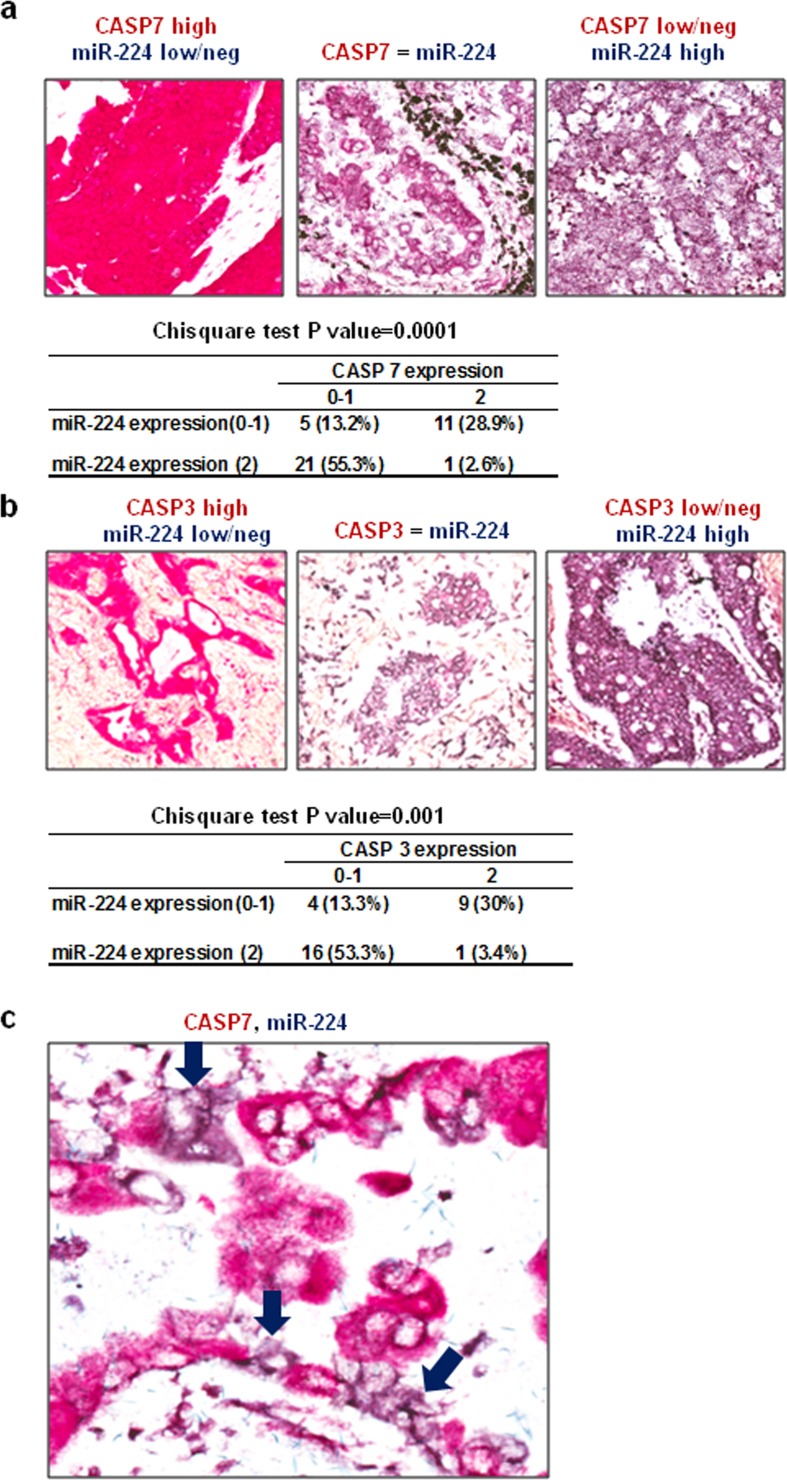
Expressions of CASP3 and CASP7 are inversely correlated with miR-224 **a.** Representative pictures and summary of co-expression analyses for miR-224 and CASP7 in NSCLC tissues. MiR-224 was detected by using 5′-DIG labeled LNA probe (purple) and CASP7 was detected by immunohistochemistry (red). Left: High CASP7 and low/neg miR-224, Middle: similar expression of CASP7 and miR-224, right: Low/neg CASP7 and high miR-224. **b.** Representative pictures and summary of co-expression analyses for miR-224 and CASP3 in NSCLC tissues. MiR-224 was detected by using 5′-DIG labeled LNA probe (purple) and CASP3 was detected by immunohistochemistry (red). Left: High CASP3 and low/neg miR-224, Middle: similar expression of CASP3 and miR-224, right: Low/neg CASP3 and high miR-224. **c.** In the cases of NSCLC where both miR-224 and CASP7 expression were noted, no detectable CASP7 was found in cancer cells overexpressing miR-224 (purple arrow).

Next, we evaluated correlation between miR-224 and its target proteins (CASP3 and CASP7) by *in situ* hybridization (ISH) analyses using 5′-DIG labeled locked nucleic acid (LNA) probe on lung cancer tissues, followed by immunohistochemistry for the target proteins. Analyzing co-expression patterns of miR-224 and CASP7 on 38 evaluable primary NSCLC tissues in human lung cancer tissue microarray revealed significant inverse correlation between miR-224 and CASP7 (*P* = 0.0001) (Figure 4a). Similar anti-correlation between miR-224 and CASP3 was also observed in evaluable 30 NSCLC tissues from OSU cohort (*P* = 0.001) (Figure 4b). Most of the lung cancer tissues showed an inverse correlation of expression between miR-224 and CASP3, CASP7, with high expression of miR-224 and low expression of CASP7 (Figure 4a) and/or CASP3 (Figure 4b) respectively, and the expressions of miR-224 and CASP7 (Figure 4c) or CASP3 (Figure S4) were basically mutually exclusive. These results suggest that reduced CASP3 and CASP7 expressions might be related to the upregulated miR-224 expression in lung cancer.

### Activated NF-κB signaling is involved in up-regulation of miR-224 in NSCLC

**Figure 5:**
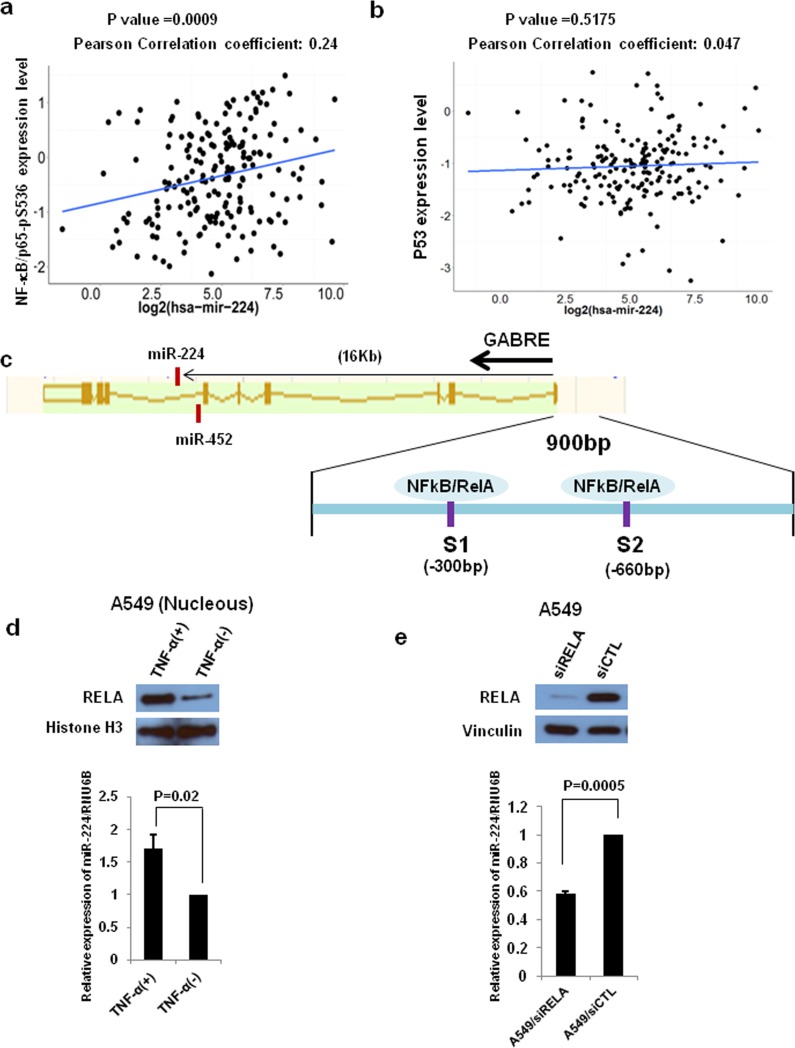
NF-kB signaling regulates miR-224 expression **a.** and **b.** NF-kB/p65-pS536 and p53 expression from TCGA RPPA data and miR-224 expression from miR-seq data were examined for correlation between miR-224 expression and NF-kB/p65-pS536 **a.** or p53 **b.** in lung ADC dataset (*n* = 188). **c.** Schematic diagram of *miR224∼miR452∼GABRE* genomic locus. MiR-224 is located in intron6 of *GABRE*. Two NF-kB/RELA binding sites were located in −300 bp and −660 bp upstream of GABRE transcription start site. **d.** Western blot analysis and qRT-PCR for A549 cells with and without TNF-α treatment to measure miR-224 expression. **e.** Western blot analysis and qRT-PCR for A549 cells with and without RELA knockdown to measure miR-224 expression. The values present mean ± S.D. as determined triplicated assays.

**Figure 6:**
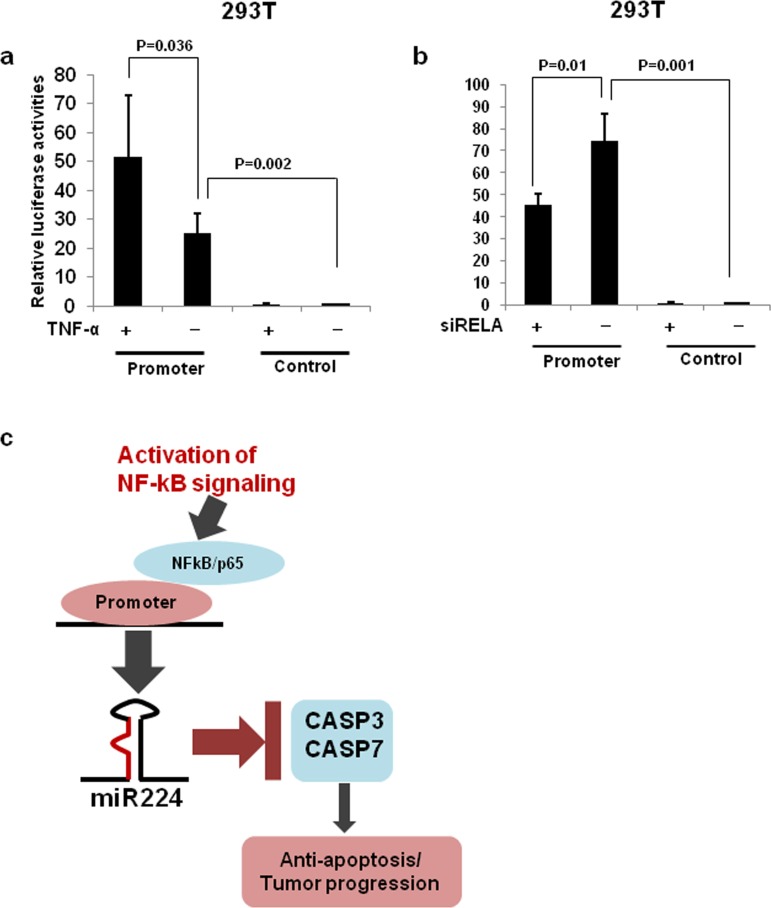
miR-224 promoter luciferase assays **a.** Luciferase reporter construct containing promoter region of miR-224 or control vector were transfected into 293T cells and treated with TNF-α for 24h. **b.** Luciferase reporter construct containing miR-224 promoter or control vector were co-transfected with si-RELA into the 293T cells. **c.** Upon activation of NFkB signaling, NFkB/p65 bind to promoter of miR-224 induces the expression of miR-224, which in turn down-regulate CASP3 and CASP7 contributing to the lung cancer progression.

Our recent study showed that miR-224 and its host gene, *GABRE*, were transcriptionally co-regulated under same promoter [10]. The p53/p65 have been reported to negatively regulate miR-224 expression by binding to its promoter region in mouse ovarian granulose cells [29]. Firstly, we analyzed the correlation between miR-224 and p53 or p65-pS536 protein expression using The Cancer Genome Atlas (TCGA) dataset. TCGA lung adenocarcinoma (ADC) (188) samples have p53 or p65-pS536 protein expression and miR-224 expression data were selected for Pearson correlation analyses. We found a significant positive correlation between p65-pS536 and miR-224 (Figure 5a), however, there is no correlation between p53 and miR-224 (Figure 5b). Since the phosphorylation of p65 at the S536 position has been associated with NF-κB/p65 activation [30] and our promoter analysis using PROMO 3.0 transcription factor binding site prediction server predicted two NF-κB/p65 binding sites in miR-224 promoter region (Figure 5c), we focused on p65 for further analyses. First, we activated NF-κB signaling by TNF-α in NSCLC cell line, A549 cells, and found the expression of miR-224 was significantly increased in A549 cells with TNF-α treatment compared to without TNF-α treatment. Enhanced RELA/p65 expression in nucleus was also confirmed in the cells with TNF-α treatment (Figure 5d). Next, we knocked down RELA/p65 in A549 cells using siRELA and analyzed its effects on miR-224 expression. Indeed, knockdown of RELA/p65 resulted in reduced miR-224 expression in lung cancer cells (Figure 5e), suggesting that NF-kb controls the expression of miR-224 in lung cancer cells. To investigate whether RELA/p65 directly binds to promoter region of miR-224 influencing its expression, we conducted luciferase promoter assays. Luciferase construct containing miR-224 promoter region markedly increased luciferase activity compared to the control vector and it was more evident after treatment with TNF-α (Figure 6a). In addition, knockdown of RELA/p65 markedly reduced luciferase activity induced by miR-224 promoter vector (Figure 6b). Altogether, these results suggest that up-regulated miR-224 expression in NSCLC might be partially controlled by NF-κB signaling through binding of RELA/p65 to miR-224 promoter region.

## DISCUSSION

MicroRNAs play crucial role in tumorigenesis [31] and miR-224 has been reported to be frequently up-regulated in a number of cancers including hepatocellular carcinoma [12, 32, 33], colorectal cancer [34], breast cancer [14] and lung cancer [10]. Our recent study demonstrated that miR-224 promotes lung cancer cell's migratory, invasive and proliferative capacity and in general tumor growth both *in vitro* and *in vivo* by direct targeting of SMAD4 and TNFAIP1 [10]. However, distinct biological functions of miR-224 were reported by Zhu et al. as they suggested high expression of miR-224 is associated with favorable prognosis in NSCLC [35]. These results are inconsistent with those results from Wang et al. as they reported that high expression of miR-224 is associated with resistance to cisplatin therapy and poor prognosis in NSCLC[36]. In fact, our recent study showed that high expression of miR-224 is associated with poor prognosis in lung ADC patients having *kras*/*p53* mutations; however, we found no association between miR-224 expressions and NSCLC patients' overall survival. These results indicate that further studies need to be done to clarify biological function of miR-224 in lung cancer.

To better understand the underlying oncogenic role of miR-224 in lung cancer, we intended to find other potential targets of miR-224. Here we report that CASP7 and CASP3 are novel direct targets of miR-224 that implicated in miR-224 induced lung cancer cell proliferation and migration.

CASP7 is an effector caspase which plays a crucial role in apoptosis [17]. The expression of CASP7 is frequently down-regulated in colorectal cancer and gastric cancer [23, 24]. Inactivating mutation in *CASP7* has been detected in colon cancer, esophageal cancer and head/neck cancer, but not in lung cancer [25]. A SNP rs2227310 in *CASP7* has been reported to be associated with risk of lung cancer and overall survival of lung cancer patients, however, whether this SNP affects protein expression in lung cancer is unknown [37]. Overall, the role of CASP7 in lung cancer remains to be elucidated. In the present study, we found that the expression of *CASP7* is significantly down-regulated in NSCLC tissues compared to NATs, and lower CASP7 expressions potentially contribute to the poor overall survival of NSCLC patients. We further demonstrated a significant inverse correlation between miR-224 and CASP7 in NSCLC tissues. Moreover, knockdown of CASP7 induced same phenotypes as the overexpression of miR-224 in lung cancer cells. These results suggest that CASP7 and miR-224 have collaborative interactions mediated lung cancer progression. In fact, different caspases have distinct role in cell proliferation. It has been reported that CASP8 and CASP6 can positively regulate B-cell proliferation; however, lack of CASP3 showed increased B cell proliferation *in vitro* [38]. In our knowledge, there is no report regarding the role of CASP7 in cell proliferation. Our study demonstrated that CASP7 negatively regulates lung cancer cell proliferation and migration. Further studies need to be done to reveal underlying mechanism of CASP7 in the proliferation and migration of lung cancer.

CASP3, a major executioner in apoptosis, is closely implicated in chemoresistance of certain type of malignancies including breast cancer [20], and colon cancer [39]. Loss of CASP3 is frequently seen in a number of solid tumors and is correlated with poor survival of patients [40-43]. In the lung cancer, low expression levels of CASP3 are associated with lymph node metastasis and worse overall survival [22]. However, somatic mutations in the coding region of *CASP3*, which cause a reduction of expression and defective function of CASP3, are infrequent [44] suggesting non-genetic alterations are involved in the down-regulation of CASP3 expression in lung cancer. In light of significant upregulation of miR-224 in NSCLC and the fact that CASP3 is a direct target of miR-224, down-regulated CASP3 might be attributable to the up-regulated miR-224 expression in NSCLC. We found that expression of *CASP3* was significantly down-regulated in NSCLC and a significant inverse correlation between miR-224 and CASP3 in NSCLC tissues. Furthermore, *in vitro* studies proved that alternation of CASP7 and CASP3 after manipulation of *miR-224* expression in lung cancer cell lines. In addition, miR-224 significantly attenuated TNF-α induced cell growth inhibition by inhibiting cleaved CASP3 and PARP1 expressions. Taken together, our findings suggest that miR-224 promotes NSCLC progression at least in part by functionally targeting CASP3 and CASP7.

Recent evidences have indicated that several signaling pathways to be involved in the regulation of miR-224. For example, hypermethylation of miR-224 promoter in prostate cancer [45], hypomethylation of miR-224 promoter and activated ERK signaling in NSCLC [10] are associated with regulation of miR-224 expression. Recently, NF-κB/p65 was reported to bind regulatory region of miR-224 within intron 6 of host gene *GABRE* promoting transcription of miR-224 in hepatocellular carcinoma [46]. Our recent study indicates that miR-224 shares the same promoter with its host gene *GABRE* in NSCLC [10], and we found that NF-κB/p65 binding sites in miR-224 promoter region. Luciferase reporter assays showed that activation of NF-κB/p65 markedly increased the luciferase activity of miR-224 promoter reporter. Inversely, knockdown of NF-κB/p65 significantly reduced the luciferase activity of the miR-224 promoter reporter, indicating alternative functional NF-κB/p65 binding sites in the miR-224 promoter region. Accordingly, our *in vitro* analyses demonstrated that increased or decreased miR-224 expressions after activation or knockdown of NF-κB/p65, respectively, in lung cancer cells. Overall, our results suggest that upregulated miR-224 expression in NSCLC might be, at least partially attributed to the binding of NF-κB/p65 to the miR-224 promoter.

In summary, we show that CASP3 and CASP7 are previously unidentified targets of miR-224, which play a crucial role in the miR-224 mediated lung cancer progression. We further show that activated NF-κB signaling is involved in the regulation of miR-224 expression in lung cancer (Figure 6c). Hence, NF-κB/miR-224/CASP3, 7 pathway might be an ideal target for therapeutic intervention in certain lung cancer patients.

## MATERIALS AND METHODS

### NSCLC tissue samples

Human Lung Tissue Microarrays (IMH-358) were purchased from Novus Biologicals, San Diego, CA. Sixty frozen tissue specimens from patients with NSCLC and thirty cases with formalin-fixed, paraffin-embedded NSCLC tissues were obtained through the OSU Comprehensive Cancer Center Tissue Procurement Shared Resource, based on The Ohio State University institutional review board (IRB) approved research protocol. We obtained written informed consent from patients before sample analyses. Tissue samples were flash-frozen using liquid nitrogen within two hours of surgical resection and stored at −80°C until analyses.

### Plasmid construction, cell lines and regents

The human pre-miRNA expression construct Lenti-miR-224 vector and human miRZip-224 anti-miR-224 miRNA construct were purchased from System biosciences. pLightSwtich empty, CASP3-3′UTR, and CASP7-3′UTR vectors were ordered from Active Motif. Mutations were generated by using the QuickChange II XL Site-Directed Mutagenesis Kit (Stratagene). shRNA control (SHC001) and shCASP7 (TRCN0000320872 and TRCN0000350297) were purchased from Sigma. Control siRNA and siRELA were purchased from Dharmacon. The cell lines used in this study were purchased from the American Type Culture Collection (ATCC). Human lung cancer cell lines H1299, H3122, H2228 and A549 were maintained in RPMI1640 medium containing 10% FBS with 100U/ml penicillin-streptomycin. The 293T cells were cultured in DMEM medium supplemented with 10% FBS and 100U/ml penicillin-streptomycin. The human TNF-α recombinant protein was purchased from Sigma-Aldrich. Antibodies against Histon H3 (D1H2), CASP7, CASP3 (8G10) and cleaved PARP1 (Asp214) were purchased from Cell signaling technology. Anti-vinculin antibody and anti-p65 antibody (C-20) were ordered from Sigma-Aldrich and Santa Cruz Biotechnology, respectively. Antibodies against CASP3 (E-8) and CASP7 (B-5) for immunohistochemical analysis were purchased from Santa Cruz Biotechnology.

### Virus infection and transfection

The pre-miR224 expression construct, miRZip-224 anti-miR-224 construct and control vector were packaged with pPACKH1 Lentivector Packaging Plasmid mix (System Biosciences) in a 293T packaging cell line. The Transdux reagent (System Bioscience) was used for virus transduction, and infected cells were selected by fluorescence-activated cell sorting (FACS) analysis (FACSCalibular, BD Bioscience). Transfection of shRNA against CASP7 was carried out with Lipofectamine LTX according to the manufacturer's instruction (Invitrogen) and transfected cells were selected by puromycin. The siRNA against RELA was transfected to cells by using Lipofectamine RNAiMAX according to the manufacturer's instruction (Invitrogen).

### Quantitative real-time PCR

Total RNAs were extracted using TRIzol Reagent (Invitrogen) according to the manufacturer's instruction. Expression of miRNA was quantified by qRT-PCR with TaqMan miRNA Reverse Transcription Kit (Applied Biosystems). Small endogenous nucleolar U6 snRNA was used as control for normalization of miRNA. TaqMan gene expression assays for CASP3 and CASP7 were purchased from Applied Biosystems to determine their expression. GAPDH was used as control for normalization of mRNA expression. cDNAs were synthesized from 3μg total RNAs using SuperScript First-Strand Synthesis System for RT-PCR (Invitrogen). All reactions were conducted in triplicates.

### Cell migration and proliferation assays

*In vitro* cell migration and proliferation assays were conducted using 8-mm micropore membranes (BD bioscience) and Cell Counting Kit8 (Dojindo), respectively, as previously described [9].

### Treatment of cells with TNF-α

The H3122 cells were treated with TNF-α and cycloheximide at final concentration of 15ng/ml and 10μg/ml, respectively. After 4h treatment, the proteins were extracted from cells and subject to western blot. The cell proliferation assay was conducted after 24 h treatment with TNF-α.

### Western blot analysis

The cells were lysed with RIPA buffer (25mM Tris-HCl (pH 7.6), 150mM NaCl, 1% NP-40, 1% sodium deoxycholate, 0.1% SDS) supplemented with Protease/Phosphatase inhibitor Cocktail (Cell Signaling Technology), and separated on 4-20% Mini-protein TGX Gels (Bio-Rad). The cytosol and nuclear proteins were extracted by using NE-PER Nuclear and Cytoplasmic extraction Regeants (Thermo). After SDS-PAGE, the proteins were electrotransferred to Immun-Blot PVDF membrane (Bio-Rad), and blocked with 5% BSA in Tris-Buffered Saline with Tween 20 (TBST) buffer. Subsequently, the membranes were incubated with primary antibody in 3% BSA in TBST, followed by incubation with appropriate horseradish peroxidase (HRP)-conjugated secondary antibody. Specific proteins were detected using the enhanced chemiluminescence system (GE Healthcare).

### TCGA dataset

The level 3 data of TCGA miRNA-sequencing (miR-seq) and TCGA reverse phase protein array (RPPA) protein expression data with clinical information were downloaded and used for analyses as described previously [10]. The correlation analyses between miR-224 and p53 or p65- pS536 expressions, Pearson correlation coefficients were calculated.

### *In situ* hybridization and Immunohistochemical analysis

Protein/microRNA co-expression analysis was carried out as previously described with minor modifications [47]. *In situ* hybridization (ISH) for miR-224 was done using the 5′ digoxigenin tagged LNA probe (Exiqon). After the ISH, we used the Benchmark LT automated system from Leica Microsystems Bondmax (Leica, Wetzlar, Germany) according to the manufacturer's specifications to perform the immunohistochemistry for CASP3 or CASP7 (Santa Cruz Biotechnology, Inc.; 1:100, antigen retrieval for 30 min). Cases were classified according to cytoplasmic miR-224 intensity as: negative = negative or faint expression in most cells; low expression = low expression in most cells or moderate expression in < 50% of the cells; high expression = moderate to strong expression in most cells. The expression of CASP 3 or CASP7 was cytoplasmic and nuclear, and expression levels for both cellular compartments were scored as for miR-224 in three classes: negative = negative or faint expression in most cells; low expression = low expression in most cells or moderate expression in < 50% of the cells; high expression = moderate to strong expression in most cells.

### Luciferase reporter assay

To determine whether miR-224 directly targets the 3′UTRs of *CASP3* and *CASP7*, 1×10^5^ 293T cells were seeded in 12-well plates overnight, then transfected with miR-224 mimic (Thermo Scientific) plus empty 3′UTR vector or 3′UTR vectors containing WT or mut-3′UTR. After 24 h, the cells were lysed and assayed using Dual Luciferase Assay (Promega) according to the manufacturer's instructions. To investigate whether NF-κB signaling is involved in the miR-224 expression, empty vector or promoter vector containing NF-κB/p65 binding sites were transfected to 293T cells. After 12h, TNF-α was added with final concentration of 15ng/ml and further incubated for 24h. To study the direct involvement of p65 on miR-224 promoter, we co-transfected siRELA pluses empty vector or promoter vector containing NF-κB/p65 binding sites to the 293T cells. After 24h, the cells were lysed and assayed using Dual Luciferase Assay (Promega) according to the manufacturer's instructions.

### Target analysis

Bioinformatics analysis was performed by using following miRNA target prediction programs: Targets can (http://www.targetscan.org/), Pictar (http://pictar.mdc-berlin.de/) and RNAhybrid (http://www.bibiserv.techfak.uni-bielefeld.de/).

### Statistical analysis

Statistical analyses were performed with the R program (version 3.0.2). Data are represented as means with standard deviation (SD) and statistical significance was determined with unpaired Student *t* tests unless indicated otherwise. *P* values less than 0.05 were considered statistically significant.

## SUPPLEMENTARY MATERIAL FIGURES


