# Supplementary material for: MicroRNA-224 is implicated in lung cancer pathogenesis through targeting caspase-3 and caspase-7
Source: Oncotarget. 2015 Aug 19;6(26):21802–15. doi: 10.18632/oncotarget.5224 (PMC4673127; doi:10.18632/oncotarget.5224)
Supplement: Supplementary file 1 [file oncotarget-06-21802-s001.pdf]

MicroRNA-224 is implicated in lung cancer pathogenesis through targeting Caspase-3 and Caspase-7

Supplementary Material

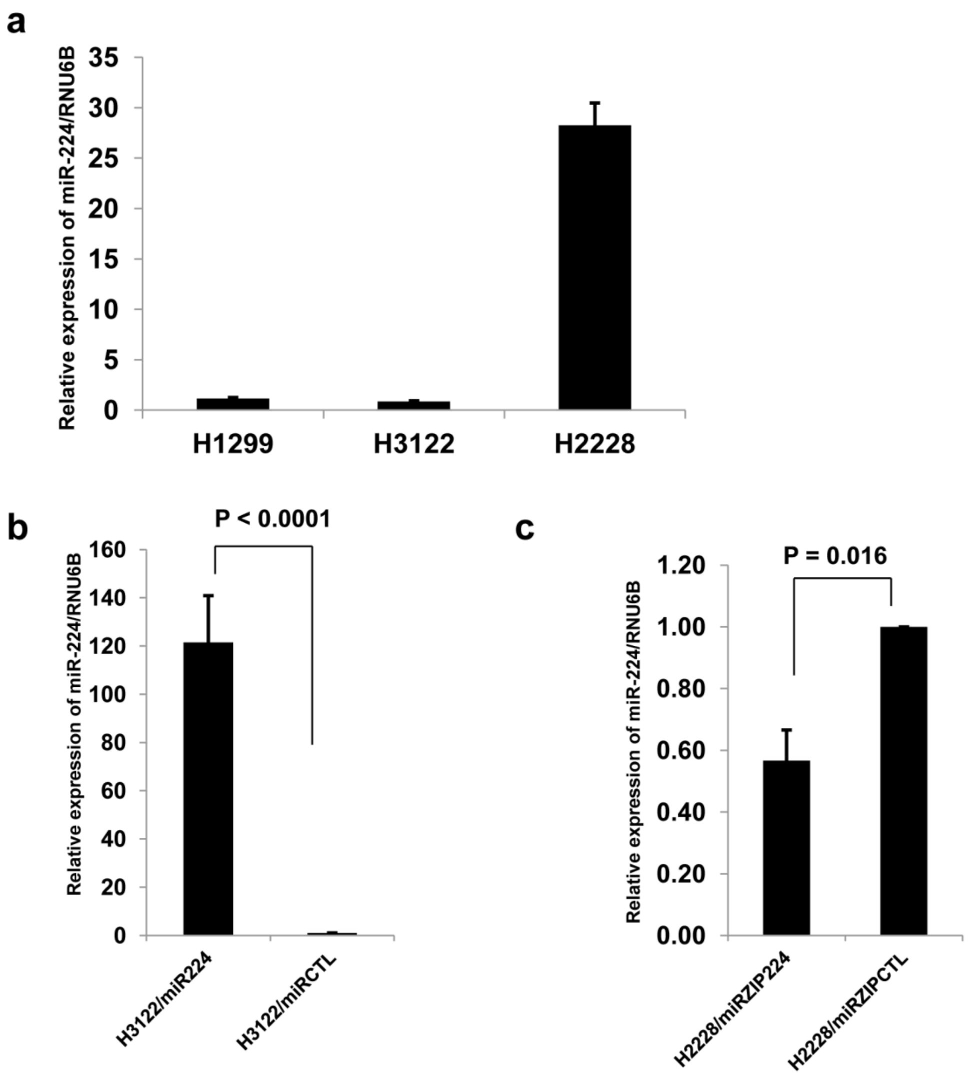

Supplementary Figure S1 a. Taqman Real-time PCR was contacted to measure the expression of miR-224 in indicated lung cancer cell lines. **b.** and **c.** miR-224 expression was measured after infection with Lenti-miR vector containing miR-224 precursor into H3122 cells (**a**) or miRZip-224 anti miR-224 construct into H2228 cells (**b**).

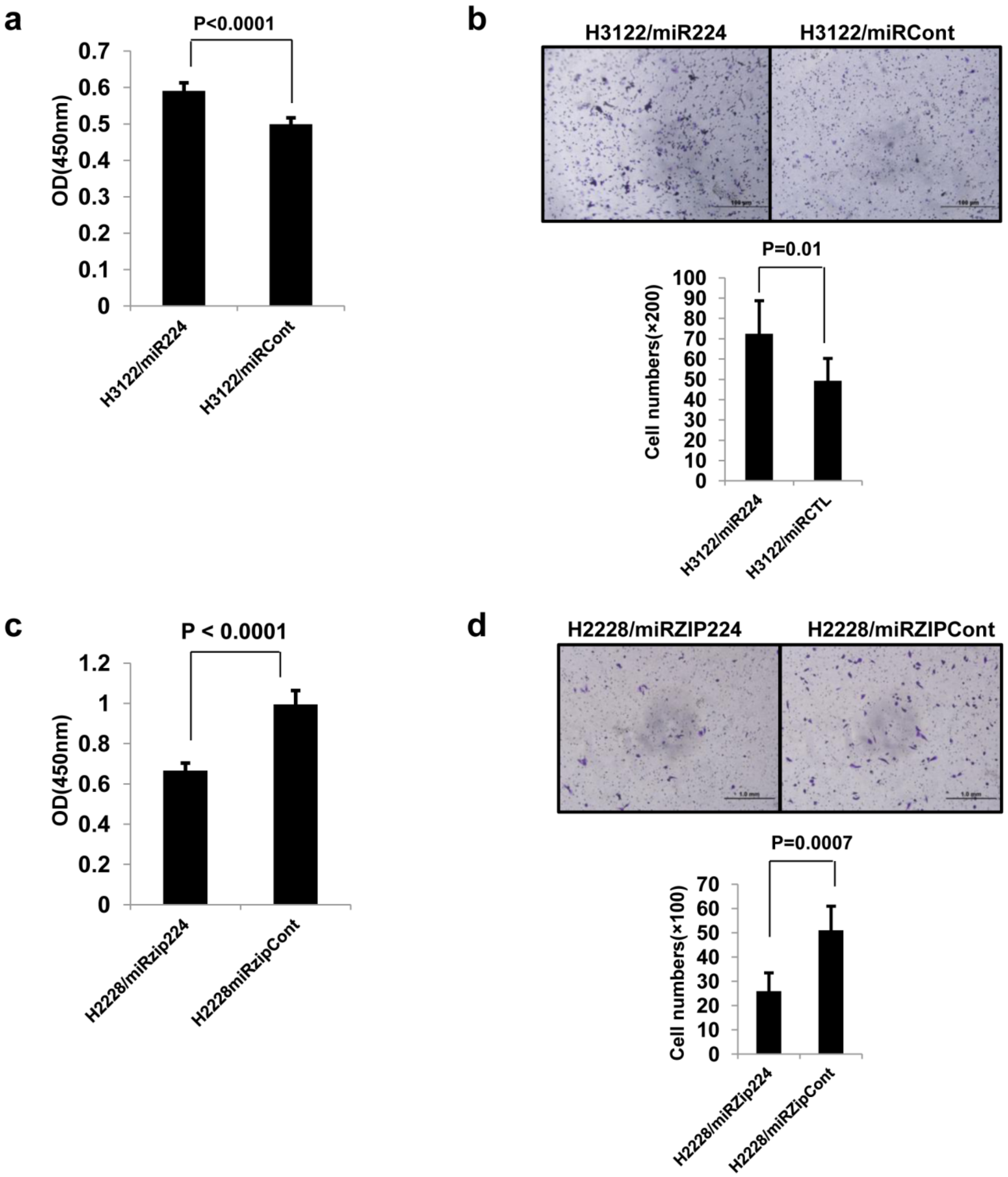

Supplementary Figure S2 a. Cell proliferation assay for miR-224 overexpressing H3122 lung cancer cells. The cell growth rates were measured by cell counting kit 8. **b.** Cell migration assay for miR-224 overexpressing H3122 cells using transwell membranes. Representative pictures of migration chambers were shown (×100 magnification).The average counts were derived from six random microscopic fields. **c.** Cell proliferation assay for miR-224 knockdown H2228 lung cancer cells. The cell growth rates were measured by cell counting kit 8. **d.** Cell migration assay for miR-224 knockdown H2228 cells using transwell membranes. Representative pictures of migration chambers were shown (×100 magnification). The average counts were derived from six random microscopic fields.

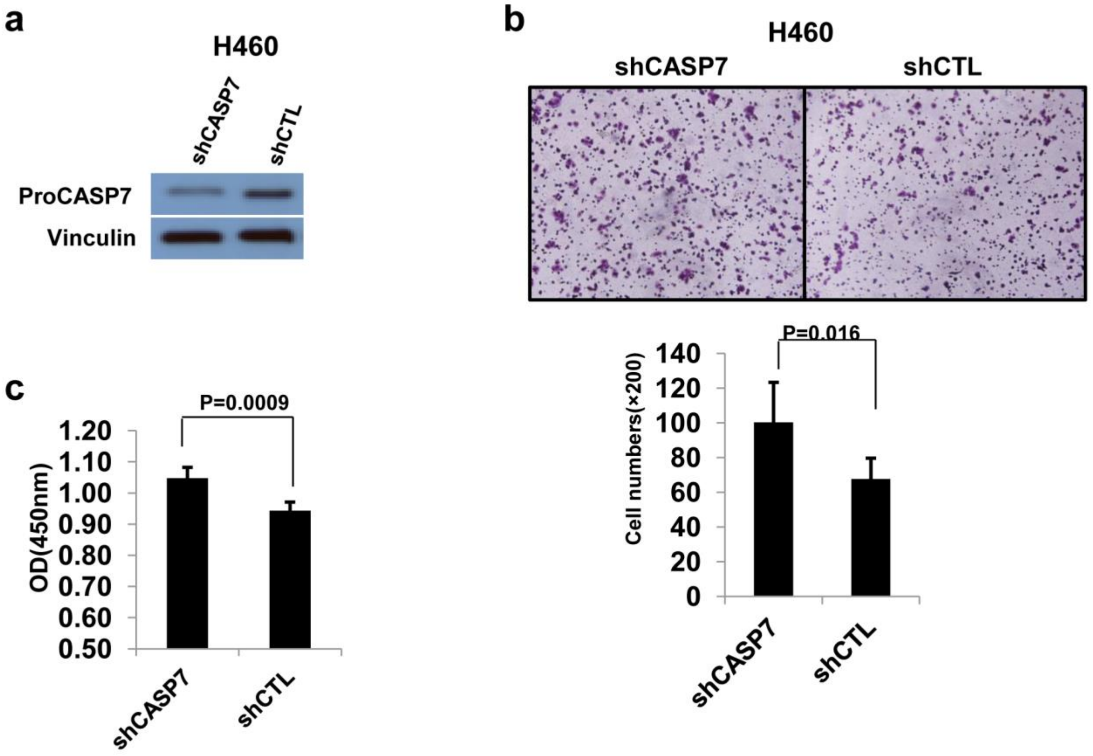

Supplementary Figure S3 a. Western blot analysis to detect CASP7 expression after knockdown of CASP7 in H460 cells. **b.** Cell migration assay for CASP7 knockdown H460 cells using transwell membranes. Representative pictures were taken under 100 times magnification. The average counts were derived from six random microscopic fields. **c.** Cell proliferation assay for CASP7 knockdown H460 cells. The cell growth late were measured by cell counting kit 8.

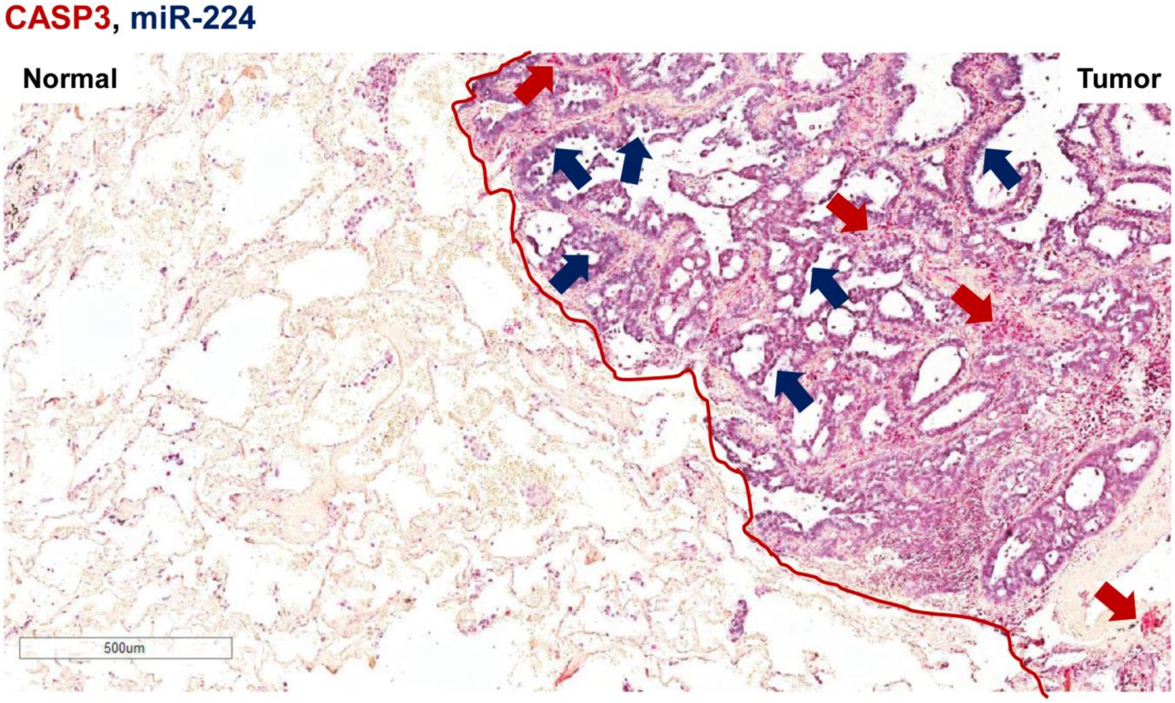

Supplementary Figure S4 Representative picture of co-expression analyses for miR-224 and CASP3 in NSCLC tissues. A red line indicates boundary of tumor tissue and normal tissue. MiR-224 was detected by using 5'-DIG labeled LNA probe (purple) and casp3 was detected by immunohistochemistry (red). In the cases of NSCLC where both miR-224 and CASP3 expression were noted, no detectable CASP3 was found in cancer cells overexpressing miR-224 (purple arrow) and vice versa (red arrow).
